# MASON CARES RANDOMIZED STUDY: EFFECTS OF A NINE-WEEK STRESS MANAGEMENT PROGRAM INTERVENTION ON CAREGIVER BURDEN SCORES

**DOI:** 10.1093/geroni/igad104.0340

**Published:** 2023-12-21

**Authors:** Gilbert Gimm, Shannon Layman, Emily Ihara, Megumi Inoue, Catherine Tompkins

**Affiliations:** George Mason University, Fairfax, Virginia, United States; George Mason University, Fairfax, Virginia, United States; George Mason University, Fairfax, Virginia, United States; George Mason University, Fairfax, Virginia, United States; George Mason University, Fairfax, Virginia, United States

## Abstract

**OBJECTIVE:**

The Mason CARES study examines the impact of a virtual stress management program (Phase 1) and music intervention (Phase 2) on the stress of family caregivers of older adults with dementia. This study presents Phase 1 findings on 99 participants enrolled in 2022.

**METHODS:**

Using primary data from 99 participants, we analyzed caregiver stress levels before and immediately after implementation of a 9-week stress management program using Zoom videoconference calls. Caregiver stress was measured using the Zarit Burden Interview (ZBI) score with a range from 0 to 48 (Bédard, 2001). Higher values (ZBI >=17.0) are associated with higher levels of stress.

**RESULTS:**

Among the 99 caregivers, 49.4% were spouses and 50.6% were non-spouses (e.g., adult children). Mean ZBI scores before and after the 9-week stress busting program intervention across the full sample decreased by 14.7% (from 23.7 to 20.2). Spouses experienced a greater decrease (17.9% from 23.5 to 19.3) in caregiver stress compared to non-spouses (12.1% from 23.9 to 21.0). Reductions in stress levels also occurred for caregivers of older adults with mild cognitive impairment (13.9% from 21.0 to 18.0), moderate dementia (16.5% from 24.1 to 20.1), and advanced dementia (9.7% from 27.7 to 25.0).

**CONCLUSION:**

Findings show that caregivers had high stress levels at baseline (ZBI score of 17 or more). Spouses reported greater stress improvement compared to non-spouses after the 9-week stress busting program. Also, caregivers of older adults with moderate dementia had greater improvement in stress compared to the mild or advanced dementia sub-groups.

